# Tracking the time course of multi-word noun phrase production with ERPs or on when (and why) *cat* is faster than *the big cat*

**DOI:** 10.3389/fpsyg.2014.00586

**Published:** 2014-07-01

**Authors:** Audrey Bürki, Marina Laganaro

**Affiliations:** ^1^Laboratory of Experimental Psycholinguistics, Faculty of Psychology, University of GenevaGeneva, Switzerland; ^2^Methodology & Data Analysis, Faculty of Psychology, University of GenevaGeneva, Switzerland

**Keywords:** language production, ERPs (event-related potentials), noun phrase production, grammatical encoding, phonological encoding

## Abstract

Words are rarely produced in isolation. Yet, our understanding of multi-word production, and especially its time course, is still rather poor. In this research, we use event-related potentials to examine the production of multi-word noun phrases in the context of overt picture naming. We track the processing costs associated with the production of these noun phrases as compared with the production of bare nouns, from picture onset to articulation. Behavioral results revealed longer naming latencies for French noun phrases with determiners and pre-nominal adjectives (D-A-N, *the big cat*) than for noun phrases with a determiner (D-N, *the cat*), or bare nouns (N, *cat*). The spatio-temporal analysis of the ERPs revealed differences in the duration of stable global electrophysiological patterns as a function of utterance format in two time windows, from ~190 to 300 ms after picture onset, and from ~530 ms after picture onset to 100 ms before articulation. These findings can be accommodated in the following model. During grammatical encoding (here from ~190 to 300 ms), the noun and adjective lemmas are accessed in parallel, followed by the selection of the gender-agreeing determiner. Phonological encoding (after ~530 ms) operates sequentially. As a consequence, the phonological encoding process is longer for longer utterances. In addition, when determiners are repeated across trials, their phonological encoding can be anticipated or primed, resulting in a shortened encoding process.

## Introduction

In recent years, research in word production has seen an increase in studies using event-related potential (ERPs) techniques. Because they allow tracking the planning process with a precision at the millisecond range, ERPs have provided a novel insight into our understanding of the language production system, and make it possible to define precise estimates of the time course of underlying processes (e.g., Costa et al., 2009; Aristei et al., 2011; Hoshino and Thierry, 2011; Laganaro and Perret, 2011; Blackford et al., 2012; Acheson and Hagoort, 2013; see also Ganushchak et al., 2011 or Strijkers and Costa, 2011; for reviews).

The large majority of these studies has focused on the production of isolated words (but see Eulitz et al., 2000; Habets et al., 2008; or Michel et al., submitted). In natural language use, however, speakers rarely utter words one by one. Even when playing the game Pictionary, where the aim is to find the one word associated with the picture being drawn as quickly as possible, speakers usually add at least a determiner. One of the main challenges of today's psycholinguistic research is to model the production process beyond single word production. Our current knowledge in this respect is still rather poor. The production of multi-word utterances is difficult to address with traditional chronometric paradigms where cognitive processes are inferred on the basis of errors or response times recorded at the end point of the production process, usually the initiation of articulation (but see Jaeger and Snider, 2013). Because ERPs monitor the on-going process, from conceptual processes to articulation, they offer a unique insight into the production of connected speech.

In the present research, we use electroencephalographic (EEG) ERPs to investigate the production of multi-word noun phrases in the context of overt picture naming. More specifically, we examine the time course differences between the production of bare nouns (e.g., *cat*), the production of noun phrases with a determiner (e.g., *the cat*, D-N), and that of adjectival noun phrases with a determiner, a prenominal adjective, and a noun (e.g., *the big cat*, henceforth D-A-N). Our aim is to pinpoint the time windows at which the encoding of the different noun phrase formats differ. We will interpret these differences in the light of (1) our current knowledge about the time course of bare noun production (2) the few proposals made to account for chronometric findings in the context of multi-word noun phrase production (e.g., Schriefers, 1992; Schriefers et al., 1999).

Psycholinguistic models usually agree that the production of speech involves three main processing stages: conceptualization, formulation, and articulation (Garrett, 1980; Levelt, 1989). During the conceptualization process, the concepts corresponding to the meaning the speaker wants to express are activated. These concepts are then sent to the formulation process where they are given a linguistic form. The formulation process takes place in three steps. The first step is called grammatical encoding. Syntactically and semantically specified lexical representations (lemmas) are selected, syntactic functions are assigned to these lemmas, and a syntactic frame is generated. In Garrett's model (e.g., 1980, see also Bock and Levelt, 1994) for instance, this results in an ordered set of word and morpheme slots. The lemmas must then be inserted in the syntactic structure. During the second step of the formalization process, termed phonological encoding, the phonological forms corresponding to the words to be uttered (lexemes) are selected and encoded. These lexemes are comprised of a set of unordered phonemes and a metrical structure (i.e., number of syllables and position of stress when relevant). The phonemes are then associated with the corresponding slots in the metrical structure during the so-called segmental spell-out. The output of the phonological encoding process is an abstract phonological word or phrase. This abstract output is fed to the phonetic encoding processing system (third step of the formulation process), where abstract articulatory gestures for each of the phonological syllables are either computed or retrieved. Once phonetic syllables have been accessed or computed, the articulation of the message can start.

During the production of multi-word utterances, speakers must activate and encode not just one but several representations at each of these levels. Moreover, in order to be fluent and produce the words in the right order, they must also master the timing of encoding of these representations, at each of these processing levels and between them. A thorough understanding of the temporal dynamics of multi-word production requires answers to at least two important questions. The first concerns the extent to which a given processing step is completed before the initiation of the next step (scope of speech planning). The second question is how these processing steps coordinate in time (temporal alignment). For instance, are lemmas and lexemes accessed and encoded in parallel, or one after the other (e.g., Lindsley, 1976; Kempen and Huijbers, 1983)? Whereas the scope of speech planning has been addressed in several studies in the past two decades, issues related to temporal alignment between and within encoding levels have been largely overlooked. In the present study, and for want of anything better, we will nevertheless consider the few proposals that have been made so far and use these proposals to make (and test) specific predictions regarding the production time course of multi-word noun phrases.

The scope of speech planning, or how much of the utterance is encoded at each processing level before the onset of articulation has been examined at the grammatical (e.g., Meyer, 1996; Smith and Wheeldon, 1999; Allum and Wheeldon, 2007; Konopka, 2012; Lee et al., 2013; Wheeldon et al., 2013) and phonological encoding levels (e.g., Schnur et al., 2006; Damian and Dumay, 2009; Martin et al., 2010; Oppermann et al., 2010; Schnur, 2011). For both encoding levels, the evidence is mixed and several authors point to flexible planning units (e.g., Martin et al., 2010; Konopka, 2012; Wheeldon et al., 2013). As concerns noun phrases, there is strong evidence that utterances comprised of a determiner, a pre-nominal adjective, and a noun (e.g., *the big cat*) or a determiner and a noun (e.g., *the cat*) are planned as a single unit at both the grammatical (see for instance Schriefers and Teruel, 1999) and phonological encoding levels (Miozzo and Caramazza, 1999; Alario and Caramazza, 2002; Costa and Caramazza, 2002; Damian and Dumay, 2007; Spalek et al., 2010; but see Michel Lange and Laganaro, 2014). Note also that the scope of planning is likely dictated in part by between-word dependencies. In French, for instance, the phonological form of the determiner as well as that of many prepositional adjectives (e.g., French adjectives *grand* “big” and *petit* “small”) depends on the noun's grammatical and phonological properties (e.g., ***le*** *grand train* “the big train” vs. ***l'****ancien train* “the old train”; *le grand camion* “the big truck” vs. *le grand(t)avion* “the big plane”). Consequently, the noun must be processed, at least to some extent, at both the grammatical and phonological processing levels, before the encoding of the determiner and adjective (see also Schriefers, 1992). In other words, French N, D-N, and D-A-N utterances can be assumed to be planned at both the grammatical and phonological encoding levels before articulation can start.

The issue of the temporal alignment of encoding processes for the constituting words of multi-word utterances also arises at both the grammatical and phonological encoding levels. At the grammatical level, the few studies converge toward the idea that lemma access for several content words usually occur in parallel (see also Dell, 1986). Moreover, there is a tendency for the speaker to wait until all slots of the syntactic frame are filled before going on to the next level. This however is not a strong constraint and speakers could variably go faster when permitted by the language syntactic dependencies, or rather, by their absence. For instance, Schriefers (1992) examined the production of A-N and D-A-N utterances in Dutch using the picture word interference paradigm. Distractors were nouns or adjectives related semantically, unrelated or identical to the target nouns and adjectives and appeared at different SOAs. He concluded in favor of an account in which lemma access for the adjective and the noun proceeds in parallel and takes longer for the noun than for the adjective (possibly because of the limited set of adjectives used in his study). Lemmas are then inserted in the syntactic frame and speakers usually wait for the last slot to be filled before going on to the next processing level (note that Kempen and Huijbers, 1983; reach the same conclusions on the basis of their experiments with short sentences, e.g., *the girl kicks*). Hence the word whose lemma access takes longer determines the length of the grammatical encoding process. Whereas this is always the case for Dutch noun phrases with definite determiners, Schriefers suggests that for A-N utterances, there may be a variable tendency for speakers to try and initiate the phonological encoding process as soon as the adjective lemma has been grammatically encoded.

Schriefers et al. (1999) also examined the production of Dutch A-N and D-A-N noun phrases and considered several accounts. In all of them, lemma access for the noun and the adjective proceeds in parallel. In these accounts the process is longer whenever two lemmas are selected in parallel as compared with one single lemma, and it does not necessarily take longer for the noun than for the adjective. In the first model, phonological encoding can start when lemma access is completed for both words. In the second account, the initiation of the phonological encoding process only waits for the completeness of the grammatical encoding process when this is necessary, as is for instance the case when the noun's grammatical gender influences the phonological form of the adjective (e.g., Dutch A-N utterances). In the third model, for D-A-N utterances, articulation is allowed to start as soon as the left most element (the determiner) is phonologically encoded (irrespective of the processing of the adjective). Data from three experiments in which participants named colored pictures while receiving advance information about the color or the object favor the second model and strongly reject the last.

Regarding the temporal alignment of the phonological encoding process for multi-word noun phrases, it is generally assumed that the activation of the different words is sequential and constrained linearly. The surface word order defined during the grammatical encoding process dictates the order in which the lexemes for the different words are encoded (see Dell, 1986; Meyer, 1996). In Meyer (1996, see also Jescheniak et al., 2003) for instance, all words receive some activation but the amount of activation decreases with the position in the utterance. In addition, the insertion of the phonemes in the metrical structure is assumed to be sequential and proceed from left to right, starting with the first phoneme of the first word (e.g., Meyer, 1990, 1991; Meyer and Schriefers, 1991; Levelt et al., 1999; Roelofs, 2004).

Given the above, we hypothesize that the production of French N (*chat*), D-N (*le chat*), and D-A-N (*le grand chat*) noun phrases will differ in the duration of both the grammatical and phonological encoding processes. Firstly, the grammatical encoding process should take longer for D-N and D-A-N utterances than for bare noun production. This is because the determiner has to be encoded in D-N and D-A-N utterances and can only be selected and inserted in the syntactic frame once the lemma for the noun has been selected. Consequently, the following process, i.e., that of phonological encoding, should start earlier for N than for D-N and D-A-N utterances. Regarding the duration of the grammatical encoding process for D-A-N vs. D-N, there are two alternative hypotheses. If the parallel activation of two lemmas takes longer than that of a single lemma (Schriefers et al., 1999), the grammatical encoding process will take longer for D-A-N than for D-N utterances. By contrast, if the duration of lemma access is determined by the word whose access takes longer (see Schriefers, 1992) there should be no difference in the length of grammatical encoding between D-N and D-A-N utterances, because lemma access will tend to be completed earlier for the adjective (most pre-nominal adjectives in French are highly frequent and should thus be accessed rapidly, especially when repeated many times in the course of an experiment) than for the noun. Secondly, the duration of the phonological encoding process (irrespective of its onset) should last longer for D-A-N than for D-N utterances, and longer for D-N utterances than for nouns produced in isolation. This is because the phonological encoding process is sequential. As a consequence, the more syllables/phonemes to be encoded, the longer the encoding process.

In the present study, we examine these hypotheses by comparing vocal response times and ERPs for French isolated nouns (N), determiner+noun (D-N), and determiner+adjective+noun (D-A-N) utterances in an overt picture naming task, with the same nouns and same participants. For response times, these hypotheses predict increasing naming times for longer noun phrases (N < D-N < D-A-N). For ERPs, they minimally predict differences between noun phrases with a determiner (D-N/D-A-N) and bare noun production at time windows associated with grammatical encoding, as well as differences between the three noun phrases (N < D-N < D-A-N) at time windows associated with phonological encoding.

In order to examine these predictions, we take advantage of ERP spatio-temporal (topographic) analyses. Previous research has shown that changes in global electric field topography over time in EEG or ERP data are not random. Rather, the signal is characterized by stable periods of electrophysiological activity at scalp, separated by abrupt changes or transition periods. This observation was first reported by Lehmann (1987), who termed the stable periods “functional microstates” (see also Koenig and Wackermann, 2009; Michel et al., 2009). During these microstates, topographic maps are highly correlated and can thus be identified with cluster analyses. Numerous studies have examined changes in the ERP signal using topographic analyses in several cognitive domains, as for instance in language processing (e.g., Koenig and Lehmann, 1996; Khateb et al., 1999; Ortigue et al., 2004) including naming data with isolated words (e.g., Laganaro et al., 2009; Laganaro and Perret, 2011). Topographic analyses allow locating the origin of response time differences between conditions as analyses can be carried out on ERP time-windows of variable duration, and covering the time interval from stimulus onset to articulation (Laganaro, 2014). For instance, Laganaro et al. (2012) examined differences between fast and slow speakers for early and late acquired words. Very slow and very fast speakers had a mean difference of 170 in naming times. The authors tracked the origin of this difference with spatio-temporal analyses and showed that a large part of this difference (90 ms) was explained by differences in the duration of an early period of topographic stability, from 200 to 350 ms after picture onset, a time window they relate to lexical selection. Another significant part (52 ms) of the difference in the behavioral responses was associated with a difference in the period of topographic stability immediately preceding articulation. Moreover, these analyses may detect differences in the duration of periods of stable global electric field that are no longer present at the end of the planning process. Here we exploit spatio-temporal analyses to examine the time course of multi-word noun phrase production as compared with that of bare noun production. Following the methodology described in Valente et al. (2012; submitted), we performed the statistical validation of the topographic analysis at the single trial level. This method has several advantages over classical ANOVAs testing for differences in conditions between participants-averaged ERPs, including the possibility to introduce covariates and to account for between speaker and between items variability.

## Materials and methods

### Participants

Twenty-seven participants took part in the study. They were all native speakers of French, right-handed (as confirmed by the Edinburgh Handedness Scale, Oldfield, 1971), and with no reported hearing or language disorders. They were all University students and were paid or given course credit for their participation. The data of six of them were disregarded due to insufficient uncontaminated ERP epochs. The 21 remaining participants (two men, mean age: 22) were included in the analyses.

### Materials

**Figure 1:**
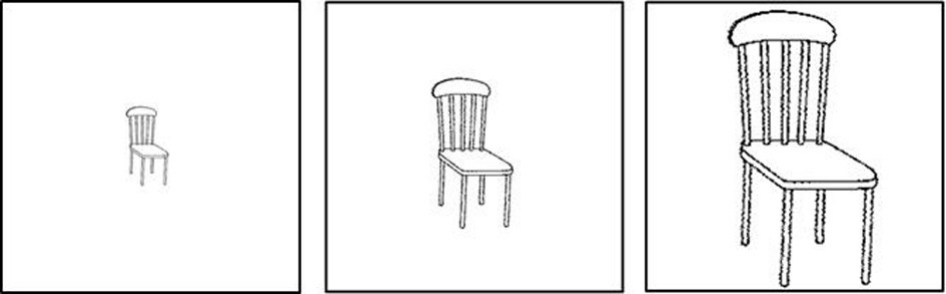
**Example of pictures used in Experiment**.

We selected 100 black and white drawings from Alario and Ferrand (1999) and Bonin et al. (2003). Fifty were early acquired words (mean age of acquisition: 1.7 on a 5 point scale), fifty were late acquired words (mean: 3.1). All nouns started with a consonant. The complete list of stimuli and their properties is given in Supplementary Materials. The following variables were available or computed for these words and used in the statistical analyses: Visual complexity, Concept familiarity, Image variability, Image agreement, Name agreement [all these measures were taken from Alario and Ferrand (1999) or from Bonin et al. (2003)]; Lexical frequency (Frantext counts), number of phonemes, number of syllables, phonological levensthein distance (following Yarkoni et al., 2008), and number of phonological neighbors. We also selected two highly frequent pre-nominal adjectives, *petit* “small” and *grand* “big.” Each picture appeared in three sizes; small, medium and large (see Figure 1) within the same size black outline rectangle (338^*^330 pixels).

### Procedure

The experiment was run with the E-prime 2.0 software (Psychology Software Tools, Schneider et al., 2002, Pittsburgh, PA). Participants were tested individually, in a sound proof booth. The experiment started with a familiarization phase. Participants were given a booklet with all the pictures (in medium size) and their corresponding nouns. They were told that they would later have to name these pictures. During the experiment, participants had to name each picture in three conditions. In one condition, the noun had to be produced in isolation (e.g., *chat* “cat”). In another condition, the noun had to be preceded by the appropriate singular definite determiner (*la* for feminine words, *le* for masculine words, e.g., *le chat* “the cat,” *la chaise* “the chair”). In the third condition, the noun had to be produced with the appropriate definite determiner (*le* or *la*), the adjective *grand* “big” or *petit* “small” depending on the size of the picture, and the noun (e.g., *la grande chaise* “the big chair”). In this last condition, half the objects were small and half were large. The size of a given object (small vs. large) was counterbalanced across participants. The three conditions were presented in different blocks, and the order of the blocks (whether participants started with the N, D-N, or D-A-N condition) was counter-balanced across participants. Picture presentation was randomized within each block.

A typical trial in the test phase had the following structure: a fixation cross was first presented in the center of the screen for 500 ms, followed by a 200 ms blank screen interval. The picture then appeared on the screen and stayed there for 1500 ms (bare noun production), 1700 ms (nouns produced with a determiner), or 2000 ms (nouns produced with a determiner and an adjective). A 2000 ms interval separated trials. Participants were told to blink rightly after having named the picture and to avoid blinking at other times. The difference in picture duration for the three utterance formats was introduced to compensate for the fact that longer sequences take more time to be articulated and possibly initiated.

### Analysis of vocal responses

Participants' responses and naming latencies (i.e., time interval between the onset of the picture presentation and the onset of articulation) were manually checked for accuracy. The item *toilette* (WC) was removed due to a high proportion of naming errors (43%). For the remaining trials, there were 523 errors for 6237 observations (8%), most of them being due to dysfluencies (42% of errors) or to the selection of the wrong noun (37%). Of the remaining errors, 4% involved the adjective in D-A-N utterances, 8% involved the determiner in D-N or D-A-N utterances, 1% involved a mispronunciation of the noun, and 6% were no responses. Eight additional data points were removed due to a difficulty in setting the vocal response onset. The analysis of response times was further restricted to the 4797 trials corresponding to valid epochs (see below).

### EEG recording and pre-processing

The EEG was recorded from 128 channels, using the Active-Two Biosemi system (BIOSEMI, Amsterdam). The signal was sampled at 512 Hz and band-pass filters were set between 0.16 and 100 Hz. The Cartool software (Brunet et al., 2011) was used for post-acquisition ERP extraction and analyses. Offline, ERPs were re-referenced against the average reference and band-pass filtered between 0.2 and 30 Hz (2nd order acausal Butterworth filter with −12 dB/octave roll-off). A notch filter was also applied, set to 50 Hz. We did not apply any baseline correction. Only epochs with correct naming responses were considered for further pre-processing. These epochs were visually checked and epochs with artifacts (eye blinks, movements) were excluded from further analysis. Epochs with amplitudes exceeding ±100 μV were also automatically rejected. 4797 epochs were included in further analyses. Electrodes with bad signals were interpolated using a 3-D spline interpolation.

For each valid trial, we extracted the stimulus-aligned epoch [from picture onset to 250 time frames (TF)- or 488 ms] and the response-aligned epoch (from −588 to −100 ms before the onset of the verbal response). Time windows of 250 TF were selected for analysis so that the combination of the stimulus-aligned and response-aligned epochs would cover the time interval from stimulus onset until 100 ms before the initiation of the vocal response. The overlapping part of the signal was then removed from the response-aligned epoch (following the approach introduced in Laganaro and Perret, 2011). This was done using the programming language python (Python Software Foundation. Python Language Reference, version 2.7 available at http://www.python.org). We also combined stimulus-aligned and response-aligned epochs averaged over trials for each participant and condition.

### ERP analysis

The aim of the ERP analysis was to compare the production time course of bare nouns, D-N and D-A-N utterances. To this end we performed a spatio-temporal segmentation on the group-averaged ERPs for each experimental condition. In the present study, and following Valente et al. (submitted), the effects of the experimental conditions were validated in the single trial ERPs.

In spatio-temporal segmentations (or topographic analyses), a spatial cluster analysis is typically used to define the dominant topographies in a given set of ERPs (i.e., determine the prototype topographic maps that best explain the data at each time frame, see for instance Michel et al., 2009) in an objective way. In this study, the spatio-temporal segmentation was performed using the TAAHC clustering algorithm (Pascual-Marqui et al., 1995; Murray et al., 2008) implemented in CARTOOL (Brunet et al., 2011). The optimal topographic map sequence for our data was determined on the basis of the ERP data averaged over all participants for the three utterance formats and two age of acquisition conditions. The selection of the best sequence of maps was based on a combination of the cross-validation criterion and the Krzanovski-Lai criterion (see Murray et al., 2008). A given topographic map had to be present for at least 10 TF (19 ms) to be retained. This criterion of minimal duration is used to eliminate transitions between two periods of topographic stability, i.e., intervals of topographic instability, as these last around 20 ms (Lehmann, 1987; Michel et al., 1999).

Visual inspection of the individual ERPs showed topographic variability in the time-window between 100 and 200 ms (see Valente et al., submitted, for evidence that the time period following the P100 in an overt picture naming task with isolated nouns is characterized by inconsistent topographies across trials). To better account for this variability, the spatio-temporal segmentation was carried out separately on the first 200 ms and from 200 ms after picture onset to 100 ms before the onset of articulation.

The topographic maps identified in the grand-averaged ERPs were then compared, time frame by time frame, with the scalp topography obtained for each single trial epoch. In this procedure, each time frame is associated with the topographic map with which it has the best spatial correlation. This procedure, called *fitting* allows determining whether a given topography is present in the single trial epoch. If present, its duration is computed as well as the percentage of variance explained by the topographic map template [i.e., global explained variance (GEV)]. The durations of the periods of topographic stability in the single trial ERPs were then used as dependent variables in the statistical analyses.

### Statistical analyses

Naming latencies (in ms) and durations (in number of TF) of periods of electrophysiological stability -topographic maps—were analyzed at the single trial level with mixed-effects regression models (e.g., Goldstein, 1987; Baayen et al., 2008, see also Valente et al., 2012; for an application of mixed-effects regression models in the context of topographic analyses) using the statistical software R (R Development Core Team, 2007, 2013). The fixed part of the model was initially comprised of utterance format (N vs. D-N vs. D-A-N), block order, repetition (whether it was the first, second or third time the participant named a given picture) and several picture- or word-related predictors known to affect naming response times: age of acquisition (i.e., age at which the noun is learned, e.g., Morrison et al., 1992), name agreement (proportion of convergent responses amongst participants for a given picture, e.g., Paivio et al., 1989; Vitkovitch and Tyrrell, 1995; Snodgrass and Yuditsky, 1996), visual complexity of the picture (Snodgrass and Vanderwart, 1980), familiarity of the concept (Snodgrass and Vanderwart, 1980), lexical frequency (e.g., Barry et al., 1997; Alario et al., 2004), word length (number of phonemes/syllables, e.g., Roelofs, 2002), phonological neighborhood (Sadat et al., 2014), image agreement (e.g., Barry et al., 1997), and image variability. We also tested for the presence of interactions between utterance format and each picture- or word-related predictor.

We checked for pairwise correlations between each pair of word- or picture-related predictors. Whenever a correlation between two predictors was above 0.3, we residualized one of the predictors. Residualization between two predictors (e.g., A and B) was performed as follows. We first ran a linear model where predictor A was predicted by predictor B. We then used the residuals of this model rather than the raw measures for predictor A. Note that in all the statistical models we present, none of the predictors needed to be residualized.

The random part of the model included random intercepts for participants and items, and random slopes allowing for the effects of the predictors to differ across participants or items for all between-unit predictors (see for instance Baayen and Milin, 2010; Barr et al., 2013). The inclusion of random slopes is required in regression models in order to ensure that the results are not driven by a restricted set of participants or items and can thus be generalized. Unless otherwise stated, all the results we report come from models with the maximum random effect structure, and where only the predictors that reached significance or were involved in a significant interaction were retained (note that in all analyses, the results are the same if non-significant predictors are retained in the models). Significance was assessed using the two following criteria: *t*-value above 1.96 for the estimate, and *p*-value from sequential F-tests based on MCMC sampling (with denominator degrees of freedom equal to the number of observations minus the number of predictors, see Baayen, 2008) equal or below 0.05. Following Baayen (2008), and in order to ensure that the results in our final models are not driven by few atypical data points, residuals larger than 2.5 times the standard deviation were considered outliers and removed.

## Results

### Response times

Mean response latency for the 4797 valid epochs was 771 ms. In the statistical model, we took the inverse of the production latencies as our dependent variable, as indicated by the Box–Cox test (Box and Cox, 1964). Results revealed longer latencies for D-A-N utterances (800 ms) than for N (756 ms) or D-N utterances (759 ms). 95% confidence intervals around the mean differences for D-A-N vs. N, D-A-N vs. DN, and D-N vs. N utterances, were respectively of [31.1–54.1], [31.9–55.7], and [−12.6–10.2]. Response latencies were shorter for early (749 ms) than for late acquired nouns (794 ms), and decreased with name agreement and repetition (with means of 714, 657, and 648 ms for the first, second, and third repetition, respectively). There was also a main effect of block order. There was no interaction between age of acquisition and utterance format (*p* = 0.2), between name agreement and utterance format (*p* = 0.9) or between utterance format and repetition (*p* = 0.2). None of the other picture- or word-related predictors influenced the production latencies or interacted with utterance format. The statistical details of this analysis are given in Table 1. There was no potentially harmful multicollinearity in this model (minimum tolerance value = 0.986). Note that due to lack of convergence, the model does not contain random terms allowing for the effects of name agreement and block order to vary amongst participants^1^.

**Table 1:** **Summary of mixed-effects regression model for response latencies**.

**Variable**	β^a^	***SE***	***t***	**(*df*)*F***	***p*-values (*F*-tests)**
Repetition (1)				(2, 4706) = 10.18	*p* < 0.0001
1 vs. 2	−8.71·10^−5^	1.83·10^−5^	−4.75		
1 vs. 3	−1.02·10^−4^	2.27·10^−5^	−4.48		
(2 vs. 3	−7.99·10^−6^	1.71·10^−5^	−0.47)		
Block order^b^				(5, 4706) = 20.59	*p* < 0.0001
Utterance format (N)				(2, 4706) = 3.85	*p* < 0.05
N vs. D-N	1.52·10^−7^	1.98·10^−5^	0.008		
N vs. D-A-N	7.10·10^−5^	2.50·10^−5^	2.85		
(D-N vs. D-A-N	6.63·10^−5^	1.86·10^−5^	3.57)		
Age of acquisition (late acquired words)	5.94·10^−5^	1.74·10^−5^	3.41	(1, 4706) = 13.79	*p* < 0.001
Name agreement	−3.01·10^−6^	7.39·10^−7^	−4.01	(1, 4706) = 16.61	*p* < 0.0001

*The intercept represents the estimated performance for the first repetition of an early acquired noun produced in isolation. For categorical variables, the statistical values correspond to the contrast between the intercept and the level of the variable indicated in parentheses*.

*N, noun; D, Determiner; A, Adjective. P-values based on MCMC sample, with denominator degrees of freedom being n (number of observations) minus the number of predictors*.

a *All the β reported in the manuscript are unstandardized*.

b *Block order was entered in all analyses in order to ensure that the effects of condition we report are not driven by differences in the order of presentation of the three utterance formats. Given that we are not interested in this variable per se and given that the six modalities of this predictor result in a complex pattern, we do not report all the pairwise comparisons for this predictor in the present or subsequent analyses*.

### ERPs

**Figure 2:**
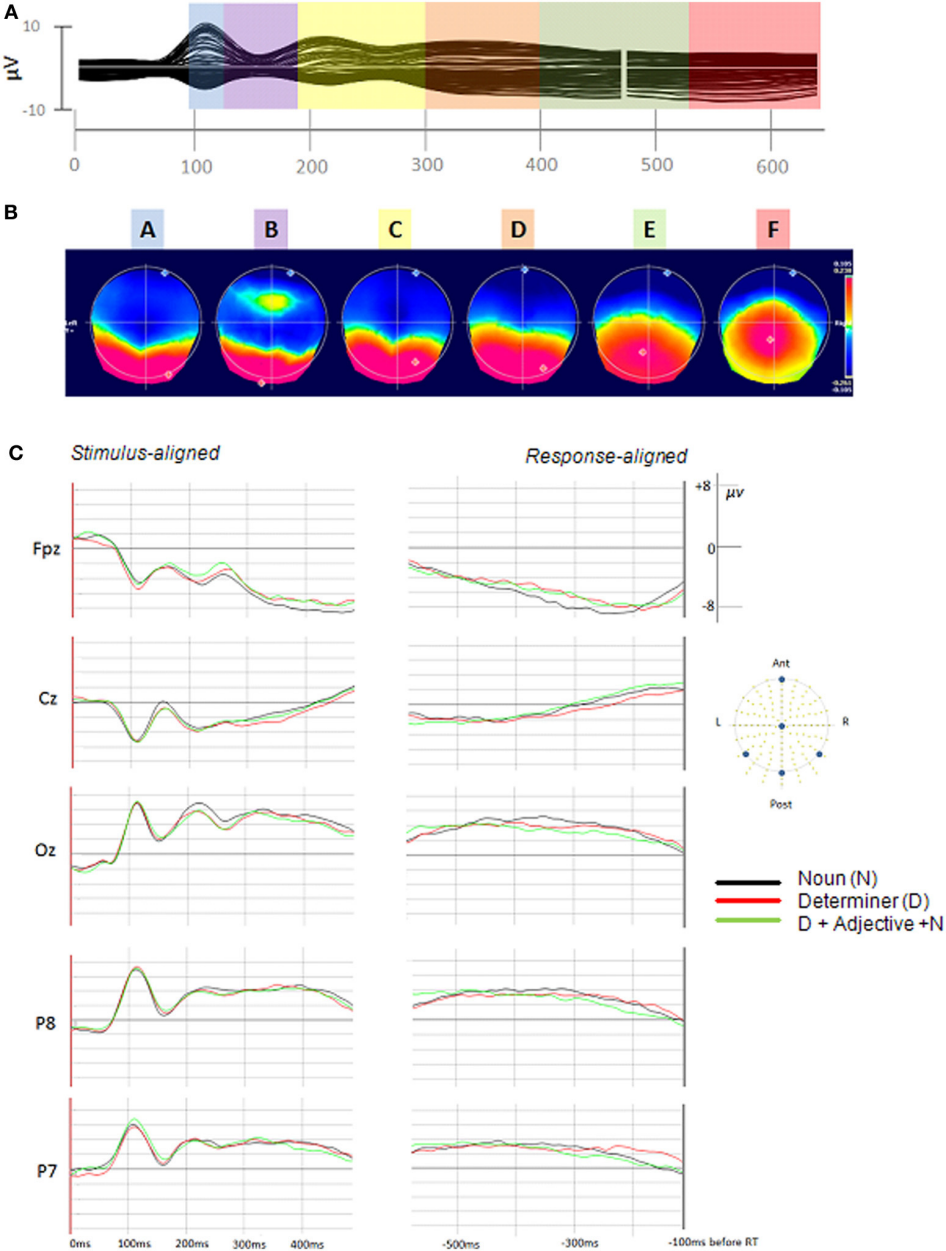
**(A)** Stimulus-aligned and response-aligned grand-average ERP. The colors summarize the durations of the periods of stable global electrophysiological patterns (topographies), maps A, B, C, D, E, and F, respectively. **(B)** The corresponding topographic map templates are represented (positive values are in red and negative values are in blue) with display of maximal and minimal scalp field potentials. **(C)** Examples of averaged stimulus-aligned and response-aligned ERP waveforms for each utterance format with the arrangement and electrode position of the displayed waveforms (Cz, Fpz, Oz, P7, and P8).

The spatial cluster analysis applied to the participant-averaged ERPs (one for each utterance format/AoA modality) resulted in four topographic maps in the first segmentation period (0–200 ms) and in four topographic maps in the second segmentation period (the first topographic map being identical to the last one of the first segmentation period). All map templates except for the one appearing in the first 50 ms were considered in further analyses (labeled A–F in Figure 2). These six topographic maps accounted for 98% of the explained variance in the grand-average ERPs. The topographic maps and their corresponding periods of topographic stability (color bars) are presented in Figure 2.

In order to determine the presence and duration of these template maps in the single trial epochs, the six maps were fitted back in the single trial epochs, from 100 ms after picture onset to 100 ms before the initiation of articulation. On the basis of the spatio-temporal segmentation obtained for the grand-averages, we used the following fitting time windows: from 50 (97 ms) to 110 TF (216 ms), from 111 (217 ms) to 250 TF (488 ms), and from 251 TF (489 ms) to the end of the epoch (100 ms before articulation onset). These time windows were selected so that they would start and end in the middle of stable electrophysiological activity (topographic maps). This was done to account for variability in the duration of each period of stability among items and participants. Topographic maps at the edge of fitting time windows (maps C and E) were entered in two consecutive fitting periods: maps A, B, and C in the first time window, maps C, D, and E in the second, and maps E, and F in the last. The durations of maps C and E were computed by adding the duration of these maps in the first fitting period to the duration of these same maps in the second fitting period.

Each map was present in at least 62% of the single epochs and the percentage of explained variance varied from 7% (map B) to 32% (map C). Table 2 summarizes the proportion of map presence in the single trials and shows the GEV for each map.

**Table 2:** **Proportion of map presence and global explained variance for each topographic map in the single trial ERPs (*n* = 4797), for each utterance format**.

	**Map A**			**Map B**			**Map C**			**Map D**			**Map E**			**Map F**		
	**(~100–130)**			**(~130–190)**			**(~190–300)**			**(~300–400)**			**(~400–530)**			**(~530–100 before articulation)**		
	N	D	A	N	D	A	N	D	A	N	D	A	N	D	A	N	D	A
% of trials with map	64	63	62	68	65	69	74/88	75/92	72/91	75	77	73	94/77	91/81	93/76	75	69	82
GEV	23	24	23	7	7	7	31/16	32/18	32/18	22	23	20	20/25	16/26	19/27	19	17	19

*GEV and proportion of map presence for maps C and E are provided for the two fitting time windows separately*.

*N, isolated noun; D, Determiner+Noun utterance; A, Determiner+Adjective+Noun utterance*.

We conducted a mixed-effects regression model to predict map duration (in number of TF, one TF being equal to 1.95 ms), considering only the trials in which a given map was indeed present. Block order, utterance format, map, repetition, and all picture- and word-related variables were entered as predictors. We also examined the two-way interactions between the factor map and each of the other predictors. This model only had random intercepts for items and participants^2^. Results of the sequential *F* tests for this model are presented in Table 3.

**Table 3:** **Summary of mixed-effects regression model for map duration (sequential *F* tests)**.

**Variable**	***F***	***DF***	***p*-values**
Block order	5.02	5, 22,241	*p* < 0.001
Map	2919.83	5, 22,241	*p* < 0.0001
Repetition	46.69	2, 22,241	*p* < 0.0001
Utterance format	24.76	2, 22,241	*p* < 0.0001
Age of acquisition	26.49	1, 22,241	*p* < 0.0001
Name agreement	22.19	1, 22,241	*p* < 0.0001
Map: block order	74.01	25, 22,241	*p* < 0.0001
Map: repetition	14.23	10, 22,241	*p* < 0.0001
Map: utterance format	17.77	10, 22,241	*p* < 0.0001
Map: age of acquisition	14.00	5, 22,241	*p* < 0.0001
Map: name agreement	19.85	5, 22,241	*p* < 0.0001

In this model, six predictors significantly predicted map duration or were involved in a significant interaction with the factor “map:” Block order, Repetition, Map, Utterance format, age of acquisition, and name agreement. There was no potentially harmful multicollinearity in this model (minimum tolerance value = 0.975). In order to better understand the role of these predictors on the duration of each map, and to be able to introduce random slopes for all these predictors, we conducted separate models for each map. In these models we also tested for the interaction between utterance format and the two lexical variables (name agreement and age of acquisition). Table 4 summarizes the results for each map.

**Table 4:**
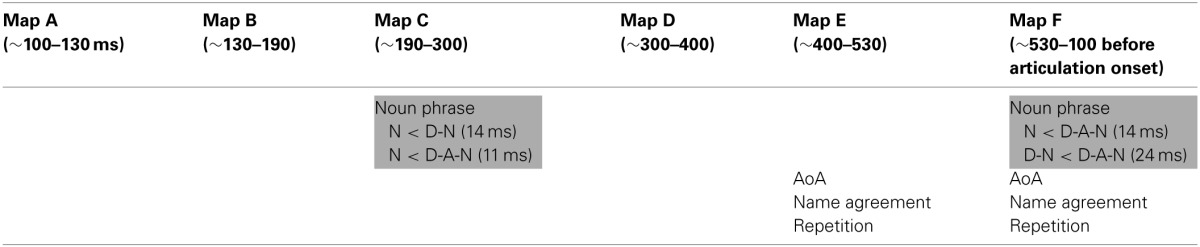
**Summary of the effects of utterance format (in grey) and of other predictors for each topographic map**.

*Differences between maps are the differences estimated by the statistical models. N, isolated noun; D-N, Determiner+Noun utterance; D-A-N, Determiner+Adjective+Noun utterance*.

The duration of map A (from about 100 to 130 ms after picture onset), B (from **~**130 to 190 ms after picture onset), and D (from **~**300 to 400 ms after picture onset) were not influenced by utterance format or any of the predictors. Utterance format did not interact with name agreement, or age of acquisition.

Map C (from about 190 to 300 ms after picture onset) was influenced by utterance format [*F*_(2, 4673)_ = 6.17, *p* < 0.01] with a shorter duration for N than for D-N utterances (β = −6.96, t = −3.07, *p* < 0.0001) and a shorter duration for N than for D-A-N utterances (β = −5.38, *t* = −2.38, *p* < 0.0001). There was no difference between D-N and D-A-N utterances (β = −1.58, t = −0.58, *p* = 0.19). None of the other variables or interaction between utterance format and the lexical variables were significant.

The duration of Map E (from about 400 to 530 ms after picture onset) decreased with name agreement [β = −0.40, t = −3.88, *F*_(1, 4625)_ = 15.02, *p* < 0.001] and was influenced by block order [*F*_(5, 4625)_ = 5.48, *p* < 0.01]. None of the other variables or interactions between utterance format and the lexical variables were significant. Note that in this model, there is no random slope allowing for the effect of block order to differ among items.

Finally, the duration of Map F (from about 530 after picture onset to 100 ms before the initiation of articulation) was influenced by utterance format [F_(2,3510)_ = 7.10, *p* < 0.001] with a longer duration for D-A-N than for N utterances (β = 7.38, t = 2.34) or D-N utterances (β = 12.55, t = 2.54, no difference between N and D-N utterances, β = 4.81, t = 1.50). The duration of map F was also influenced by block order [F_(5, 3510)_ = 23.42, *p* < 0.0001], and by repetition [F_(2, 3510)_ = 5.11, *p* < 0.01], with a longer map for the first repetition than for repetition 2 (β = 9.67, t = 2.83) or repetition 3 (β = 6.32, t = 2.11) but no difference between repetitions 2 and 3 (β = 3.46, t = 1.43). Map F was also longer for late than for early acquired words [β = 8.69, t = 3.49, *F*_(1,3510)_ = 16.28, *p* < 0.0001] and decreased with higher name agreement values [β = −0.36, t = −3.17, *F*_(1, 3510)_ = 10.04, *p* < 0.01]. No other variable influenced the duration of Map F and the lexical variables did not interact with utterance format for this map.

## General discussion

In this study, we examined the temporal dynamics of multi-word utterance production. We compared the production of bare nouns (e.g., *cat*), noun phrases with a determiner (e.g., *the cat*, D-N), and noun phrases with a determiner, a prenominal adjective, and a noun (e.g., *the big cat*, D-A-N) in a picture naming task. We recorded the participants' vocal responses, their response times and their electroencephalogram during the task. On the basis of previous proposals, we expected response times to be shorter for bare nouns than for D-N noun phrases and shorter for D-N than for D-A-N utterances. In the ERP topographic analysis, we expected to find evidence suggesting (1) shorter periods of topographic stability for bare nouns than for utterances with a determiner in time-windows associated with grammatical encoding processes and (2) longer durations for longer utterances (N < D-N < D-A-N) in time windows compatible with post-lexical phonological processes.

Behavioral analyses revealed a main effect of utterance format on naming latencies, with longer naming times for noun phrases with a determiner and an adjective than for D-N or N utterances. Contrary to our predictions, there was no difference between bare noun production and D-N utterances. These results are discussed below, together with the results of the ERP analysis.

### Early effect of utterance format

An influence of utterance format was first found at an early time window. The topographic map lasting from about 190 to 300 ms after picture onset was shorter for bare noun production than for D-N (14 ms according to the statistical model) and D-A-N utterances (11 ms). In previous estimations of the time course of the processing stages underlying bare noun production, similar time windows have been associated with grammatical encoding processes (e.g., Strijkers et al., 2010; Indefrey, 2011). According to Indefrey for instance, lemma retrieval starts at ~200 ms and ends before 270–290 ms after picture onset. The early ERP difference we observe between noun phrases are thus likely to reflect operations taking place during grammatical encoding processes. Note also that the finding that this time window is modulated by the presence of a determiner can be taken as an additional argument that this time window overlaps with grammatical encoding processes.

As discussed in the Introduction, within a unit of grammatical encoding, lemma access for the different words is thought to occur in parallel (Kempen and Huijbers, 1983; Schriefers, 1992; Schriefers et al., 1999) except in the case of syntactic dependencies. For instance, when the gender of the determiner depends on the gender of the noun (as is the case for many French determiners), lemma access for the determiner can only start once the noun lemma has been selected (Schriefers, 1992). Moreover, the phonological encoding of the utterance is only initiated once all lemmas have been inserted in the corresponding slots of the syntactic structure. In this context, the longer duration for the stable topographic pattern from about 190 to 300 ms after picture onset for noun phrases with determiners (D-N and D-A-N utterances) could reflect the additional time required to select the determiner lemma, once lemma selection for the noun (and adjective) has taken place.

The absence of difference between D-N and D-A-N utterances is as expected if lemma access for the adjective and the noun process in parallel, with the length of this process being determined by the noun (but see Schriefers et al., 1999, for the proposal that lemma access takes longer when more lemmas are selected). According to Schriefers (1992) the duration of the grammatical encoding process is determined by the word whose lemma retrieval takes longer. In the present experiment, we used visually salient and highly frequent adjectives. Moreover, the same two adjectives were repeated over and over in the course of the experiment. The adjective lemma was likely highly primed and more readily available than the noun lemma. Consequently, the duration of the grammatical encoding process likely equaled the duration of lemma retrieval for the noun, and was thus equivalent for D-N and D-A-N utterances.

In addition (or alternatively), the longer duration of the topographic map lasting from 190 to 300 ms after picture onset for D-N and D-A-N utterances than for N utterances could reflect the retrieval of additional grammatical information (gender) for D-N and D-A-N utterances, or the generation of a syntactic structure, a step that is not required in bare noun naming. According to Schriefers (1992) however, in experiments where the same syntactic structure is used in successive trials within the same block, the syntactic frame does not need to be generated on each trial.

The question may arise of whether, if the stable topographic pattern from 190 to 300 ms after picture onset indeed covers part of the grammatical encoding process, we shouldn't expect other linguistic variables to affect its duration. One variable in particular, i.e., lexical frequency, has been strongly linked with grammatical encoding. For instance, Strijkers et al. (2010) found diverging ERPs for high and low frequency words starting at about 150–200 ms after picture onset and relate this effect to lexical (lemma) access. In the present study, lexical frequency did not influence naming times or the duration of any period of topographic stability. Importantly, however, and unlike in Strijkers et al. lexical frequency was not dichotomized in our design but entered in the analysis as a continuous variable. Effects of lexical frequency on naming times usually surface in categorical design where the difference between high and low frequency words is maximized, or in naming studies with many different pictures. As for the other lexical variables we tested in the present study, none of them has been strongly and uniquely associated with lemma access. That we do not find influences of lexical variables on the third topographic map thus does no challenge the hypothesis that this map overlaps with grammatical encoding processes.

### Late effect of utterance format

Utterance format also influenced the duration of the last period of stable electrophysiological activity, from ~530 ms after picture onset to 100 ms before the initiation of articulation (end of the analyzed period). This period of stable global electric field was longer for D-A-N than for D-N (24 ms) or N utterances (14 ms). Based on current estimates of the time course of bare noun production, it is reasonable to assume that this time window covers at least part of the post-lexical phonological encoding process. In Indefrey (2011) estimations for isolated nouns and based on a mean response time of 600 ms, post-lexical phonological processes (segmental spell-out and syllabification) start around 355–455 ms after picture onset, and last about 55 ms per syllable (e.g., between 100 and 120 ms for disyllabic words with 5–6 phonemes). The onset of phonetic encoding processes (upper boundary) is estimated at about 145 ms before the initiation of the vocal response. Considering the longer naming times (about 770 ms) and the higher number of syllables (mean of 2.73) in the present study, phonological encoding processes can be expected to be slightly delayed and to last slightly longer than in Indefrey's estimates. The interval from 530 ms after picture onset to 100 ms before the onset of articulation is thus a reasonable time window for at least part of these processes.

According to current views in the literature (e.g., Meyer, 1990, 1991; Roelofs, 2004), the phonological encoding process is sequential and thus takes longer for each additional phoneme/syllable. The longer duration of the last stable topographic pattern for longer utterances (D-A-N) could thus reflect the fact that more phonemes/syllables are encoded in these sequences than in D-N and N utterances.

According to this view, the phonological encoding of D-N utterances should also last longer (about 55 ms according to Indefrey, 2011) than that of N utterances. Contrary to this prediction, there was no difference in the duration of the last topographic map between D-N and N utterances. A possible explanation for this result, as well as for the absence of difference in naming times between D-N and N utterances may reside in our experimental design. Because we used a blocked design, utterance format did not vary from trial to trial within each block. As a consequence, part of the utterance was likely anticipated and/or primed in D-N and D-A-N utterances, but this was not the case in bare noun naming. In utterances with a determiner, the phonological onset of each trial was the phoneme/l/ in all trials and its phonological encoding could thus easily be anticipated. Moreover, the second phoneme of the determiner was repeated in half the trials, as was the adjective in D-A-N utterances. Anticipation and priming may have given D-N and D-A-N utterances an advantage over bare noun production in that it reduced the duration of the phonological encoding process. In the case of D-N utterances, this difference may have counterbalanced the initial advantage of N utterances over D-N utterances. Interestingly, the ERP analysis provides evidence that the repetition of a given word across trials indeed influences the last period of stable electrophysiological topography. We find that the duration of this last stable topographic pattern decreases with the second and third repetitions of the noun. Note also that a similar absence of difference between isolated nouns and nouns preceded by a determiner has been reported in other chronometric studies with similar designs (e.g., Jescheniak et al., 2003, see also Miozzo and Caramazza, 1999).

In addition to being influenced by utterance format, the last period of stable topography was influenced by name agreement, age of acquisition and repetition. All three variables have been found to influence naming latencies in previous studies (name agreement: Vitkovitch and Tyrrell, 1995; Snodgrass and Yuditsky, 1996; Barry et al., 1997; Alario et al., 2004, AoA: Barry et al., 1997; Morrison and Ellis, 2000; Bonin et al., 2002; Alario et al., 2004, repetition: Mitchell and Brown, 1988; Wheeldon and Monsell, 1992; Barry et al., 2001). The exact locus of these effects is still debated. For instance, for name agreement, object recognition processes (but see Vitkovitch and Tyrrell, 1995), semantic processing (Kan and Thompson-Schill, 2004), and lexeme retrieval and/or phonological encoding (Johnson et al., 1996; Cheng et al., 2010) have been proposed. Similarly for AoA effects, object recognition (Catling et al., 2008), the link between visual and semantic processing (Urooj et al., 2014), the conceptual or lemma level (Belke et al., 2005; Brysbaert and Ghyselinck, 2006), lexical-phonological retrieval and/or phonological encoding processes (Barry et al., 2001; Kittredge et al., 2008; Laganaro and Perret, 2011; Laganaro et al., 2012; Navarrete et al., 2013) have been considered. Finally, the effect of repetition on naming times has been related to the retrieval of the word's phonological form (Barry et al., 2001), to semantic processes, or to the mapping of semantic features to phonological representations (Wheeldon and Monsell, 1992).

To summarize, with respect to the main research question of the present study, we conclude that the time course of encoding of N, D-N and D-A-N utterances for production differs at two time windows. Considering our current knowledge about the time course of word production, these time windows likely correspond to the grammatical and phonological encoding processes. We have argued that these differences could translate, respectively the selection of the gender-agreeing determiner during the grammatical encoding of D-N and D-A-N utterances and the phonological encoding of additional syllables/words in longer utterances.

Moreover, the results of the present study show one more time that the origin of differences in response times can be traced back using topographic analyses. Naming times were longer for D-A-N than for D-N and N utterances and according to the ERP analysis, part of this difference can be attributed to the time window associated with the phonological encoding process. In addition, topographic analyses allow detecting differences that do not surface in response times. For instance, because of the likely anticipation of the determiner, the difference between N and D-N utterances in the duration of the third period of stable topography did not surface in the naming latencies.

### Future directions

In the present study, we made and tested a number of predictions regarding the production of multi-word noun phrases, following the few previous proposals available in the chronometric literature. Naming responses and topographic analyses are surprisingly well in line with these predictions. It would however be premature to conclude that these proposals provide an accurate description of the time course of multi-word noun phrase production.

As discussed in the Introduction, the temporal dynamics of multi-word production is a complex matter. Models of multi-word production must describe and legitimate assumptions regarding several issues, including the scope of grammatical and phonological planning, and the temporal alignment (i.e., parallel vs. sequential processing) of encoding processes within and between processing stages. The present results are in line with a certain configuration of assumptions. It remains to be shown whether alternative configurations lead to discriminative predictions, and if so, these predictions should be tested.

Moreover, another important goal for further studies should be to compare the temporal dynamics of grammatical and phonological encoding processes for languages with and without syntactic and phonological dependencies and in mixed rather than blocked designs. In the present study we followed the methodology used in previous chronometric studies on similar issues and had participants produce the different utterance formats in different blocks (e.g., Schriefers, 1992; Schriefers et al., 1999). The pattern of results we obtain is likely influenced, at least in part, by this methodological choice. In particular, we argued that participants could anticipate the phonological encoding of the first phoneme of the utterance in D-N and D-A-N utterances, explaining the absence of difference in naming times and in the duration of the last stable electrophysiological pattern between D-N and N utterances. This explanation should now be tested directly by comparing the ERP pattern in blocked vs. mixed designs.

## Conclusion

The present study took on the challenge to examine the production of multi-word noun phrases with ERPs. The findings reveal that the production of multi-word noun phrases involves additional processing at two time windows, the first being modulated by the presence/absence of a determiner, the second being influenced by utterance length. Considering our current knowledge about the time course of word production with isolated word, these time windows can be related to grammatical and phonological encoding processes. More generally, the present study shows that ERPs are likely to offer a powerful insight into the temporal dynamics of multi-word utterance production.

## Conflict of interest statement

The authors declare that the research was conducted in the absence of any commercial or financial relationships that could be construed as a potential conflict of interest.

## Footnotes

^1^In order to ensure that the effects of name agreement and block order generalize to all participants, we performed additional analyses in which we removed other random slopes and included a random term allowing for the effect of name agreement or block order to vary amongst participants. In these models, the effects of name agreement and block order are still significant.

^2^Models with interactions in the random structure or with random slopes for all predictors did not converge. Note that all main effects and interaction remain significant in separate models where random slope terms are introduced for one predictor at a time.
