# Supplementary material for: Tracking the time course of multi-word noun phrase production with ERPs or on when (and why) cat is faster than the big cat
Source: Front Psychol. 2014 Jul 1;5:586. doi: 10.3389/fpsyg.2014.00586 (PMC4077314; doi:10.3389/fpsyg.2014.00586)
Supplement: Supplementary file 1 [file DataSheet1.PDF]

Appendix A

Nouns/pictures used in Experiment and their properties

Noun	AoA	NA	IA	VC	IV	CM	F	Nb of phonemes	Nb of syllables	PND	Gender
baignoire	early	100.00	4.15	2.20	2.85	4.20	7.19	6	2	1	f
balai	early	93.48	3.77	2.28	2.60	4.10	7.71	4	2	27	m
banane	early	100.00	4.60	1.21	2.23	3.87	2.45	5	2	3	f
biberon	early	100.00	4.60	2.35	3.20	1.00	3.16	5	2	0	m
boîte	early	80.43	2.70	1.21	3.87	2.97	58.77	4	1	9	f
bonnet	early	90.00	4.15	3.25	2.80	2.95	10.87	4	2	17	m
bouée	early	100.00	3.30	2.95	2.85	2.75	2.52	3	1	14	f
bougie	early	91.30	4.03	2.45	3.43	3.60	10.65	4	2	10	f
camion	early	100.00	2.77	2.90	3.29	3.23	18.19	5	2	3	m
canard	early	93.48	3.47	2.97	2.83	2.50	9.58	5	2	12	m
carotte	early	97.83	4.47	3.07	2.30	3.90	2.48	5	2	13	f
chaise	early	97.83	3.13	2.24	3.43	4.93	48.45	3	1	16	f
chapeau	early	100.00	2.93	2.38	4.00	2.83	42.48	4	2	8	m
château	early	100.00	3.20	4.05	3.55	1.90	46.16	4	2	14	m
citron	early	100.00	4.83	1.72	2.53	3.63	8.06	5	2	11	m
clown	early	95.65	3.47	4.41	2.93	1.83	4.42	4	1	6	m
cochon	early	100.00	3.60	3.21	2.43	1.83	9.06	4	2	15	m
collier	early	100.00	3.90	1.79	3.77	3.33	8.97	5	2	10	m
coq	early	78.26	4.43	3.76	2.43	2.40	10.81	3	1	30	m
cube	early	100.00	4.65	1.20	2.35	2.65	6.42	3	1	6	m
cuillère	early	95.65	3.83	2.38	2.97	4.93	4.84	6	2	2	f
fraise	early	89.13	3.03	2.76	2.70	3.20	2.71	4	1	10	f
grenouille	early	80.43	4.17	3.69	2.67	1.87	6.00	6	2	1	f
jupe	early	82.61	2.37	1.66	3.73	3.23	18.13	3	1	11	f
lapin	early	100.00	4.07	3.14	2.90	2.67	10.42	4	2	12	m
lion	early	95.65	3.53	4.17	2.33	1.50	16.94	3	1	14	m
lune	early	97.83	3.60	1.03	3.20	3.80	52.45	3	1	11	f
moustache	early	85.00	4.00	2.90	1.95	2.85	15.81	6	2	0	f
moustique	early	60.00	3.95	3.60	2.50	2.85	0.94	6	2	3	m
mouton	early	67.39	2.90	3.59	2.43	1.83	11.10	4	2	14	m
nuage	early	86.96	2.90	2.45	3.47	3.87	19.29	4	2	1	m
panier	early	97.83	2.63	4.59	3.13	2.30	16.32	5	2	8	m
pantalon	early	100.00	3.40	2.28	3.70	4.87	29.26	6	3	0	m
papillon	early	93.48	4.37	4.10	3.57	2.33	13.03	6	3	1	m
parapluie	early	100.00	3.23	3.24	3.07	3.43	6.65	8	3	0	m
pelle	early	95.00	4.55	2.85	2.30	3.05	6.55	3	1	26	f
poche	early	100.00	2.90	1.20	2.35	3.40	58.26	3	1	20	f
poire	early	93.48	4.40	1.14	2.50	3.37	6.42	4	1	14	f
pomme	early	100.00	4.00	1.55	3.23	4.40	26.45	3	1	21	f
pouce	early	73.91	4.27	2.24	2.46	4.77	17.87	3	1	19	m
poupée	early	97.83	2.00	4.31	3.83	3.07	10.90	4	2	14	f

sapin	early	100.00	4.20	1.45	3.80	3.80	7.23	4	2	9	m
singe	early	93.48	3.03	3.41	3.37	1.40	10.39	3	1	7	m
souris	early	84.78	4.27	3.38	2.63	2.27	26.03	4	2	14	f
téléphone	early	100.00	3.20	3.10	3.75	4.93	60.19	7	3	0	m
tirelire	early	100.00	4.25	2.30	3.00	3.50	1.26	6	2	0	f
toilette	early	55.00	4.45	1.85	3.35	4.95	19.58	6	2	1	f
tortue	early	100.00	4.10	3.28	2.71	2.03	4.35	5	2	2	f
vache	early	89.13	3.40	3.59	2.73	2.63	18.45	3	1	19	f
vélo	early	89.13	4.20	4.17	3.57	3.37	13.00	4	2	7	m
balance	late	100.00	3.13	3.28	2.97	1.93	22.65	5	2	2	f
béret	late	90.00	3.85	3.60	2.10	2.75	6.74	4	2	9	m
bocal	late	67.39	2.83	2.66	2.97	2.93	3.06	5	2	7	m
bombe	late	95.00	3.55	1.70	2.90	2.45	15.32	3	1	8	f
borne	late	95.00	4.35	2.55	1.80	4.55	11.94	4	1	11	f
boussole	late	80.43	3.90	3.10	2.27	2.07	3.16	5	2	0	f
briquet	late	100.00	4.30	1.75	2.65	3.95	7.58	5	2	12	m
bureau	late	71.74	3.20	2.97	3.67	4.60	97.77	4	2	10	m
cadenas	late	91.30	4.20	2.14	2.47	2.47	1.42	5	2	1	m
canne	late	95.00	4.40	1.15	2.65	2.60	16.58	3	1	27	f
canoë	late	45.00	4.10	2.80	2.45	2.05	1.29	5	3	3	m
canon	late	95.65	3.43	4.62	2.03	1.07	20.81	4	2	11	m
casque	late	97.83	2.20	1.45	3.37	2.80	12.03	4	1	8	m
cercueil	late	95.00	4.55	2.00	2.90	2.75	10.74	6	2	1	m
cerveau	late	97.83	3.37	3.34	2.83	2.80	28.23	5	2	9	m
chaîne	late	91.30	2.93	2.72	3.17	2.93	45.71	3	1	22	f
cigare	late	95.65	3.80	3.59	2.33	2.00	9.55	5	2	5	m
classeur	late	100.00	4.65	2.05	2.80	4.65	0.97	6	2	4	m
commode	late	76.09	3.30	2.83	3.07	4.27	23.45	5	2	2	f
coupe	late	95.00	3.00	2.35	2.80	2.25	46.23	3	1	19	f
flèche	late	97.83	3.83	1.93	2.80	1.53	14.10	4	1	9	f
gland	late	65.22	3.37	3.38	2.11	1.90	1.84	3	1	14	m
journal	late	100.00	4.00	3.80	3.00	4.40	89.71	6	2	1	m
labyrinthe	late	95.00	4.40	3.45	2.70	2.05	5.84	7	3	0	m
louche	late	89.13	4.30	2.10	2.23	3.43	6.32	3	1	18	f
loupe	late	95.00	4.55	1.95	1.80	3.05	4.77	3	1	17	f
médaille	late	100.00	3.90	1.95	3.10	1.95	7.26	5	2	0	f
micro	late	100.00	4.70	2.50	2.50	3.35	9.94	5	2	2	m
momie	late	90.00	3.10	4.20	2.50	2.00	1.13	4	2	9	f
note	late	95.00	3.90	2.95	3.60	2.90	48.16	3	1	22	f
palme	late	95.00	4.30	1.90	2.15	2.25	2.10	4	1	7	f
palmier	late	95.65	3.83	3.76	2.77	2.10	2.55	6	2	0	m
paume	late	95.00	1.10	2.10	1.85	4.60	13.58	3	1	21	f
perceuse	late	100.00	4.30	1.40	1.70	2.95	0.19	6	2	3	f
pingouin	late	95.65	3.27	2.66	2.17	1.37	0.84	5	2	0	m
poêle	late	69.57	2.67	1.93	2.50	4.00	9.68	4	1	9	f
poignée	late	71.74	1.87	3.10	1.93	4.07	20.94	5	2	8	f

poireau	late	85.00	4.40	3.35	2.05	4.00	0.55	5	2	3	m
prise	late	86.96	1.83	2.55	2.23	3.60	84.55	4	1	14	f
pyramide	late	100.00	4.50	2.34	2.87	1.67	5.29	7	3	0	f
quille	late	100.00	4.95	1.10	2.05	2.30	2.19	3	1	17	f
sac	late	78.26	2.80	2.86	3.03	4.37	67.32	3	1	28	m
saxophone	late	76.09	4.37	4.45	2.50	1.90	0.68	8	3	0	m
squelette	late	97.83	4.77	4.66	3.00	1.60	8.52	6	2	0	m
tente	late	100.00	4.90	1.40	3.15	3.05	24.52	3	1	24	f
tonneau	late	80.43	4.57	4.10	2.33	1.27	4.10	4	2	12	m
totem	late	84.78	4.17	4.00	2.50	1.20	3.06	5	2	0	m
trombone	late	95.00	4.80	1.75	1.80	3.80	0.42	6	2	3	m
volant	late	100.00	3.75	1.90	2.60	4.35	26.81	4	2	13	m

AoA: Age of acquisition; NA: Name agreement; IA: Image agreement; VC: Visual complexity; IV: Image variability; CF: Concept familiarity; F: Frequency; PND: Phonological neighborhood density; Gender: Grammatical gender.
